# Routine C-Reactive Protein on Admission and In-Hospital Outcomes in Acute Ischemic Stroke Patients Treated with Thrombectomy: A Retrospective Single-Center Study

**DOI:** 10.3390/medicina62081462

**Published:** 2026-07-28

**Authors:** Saulius Taroza, Dalius Jatužis, Marius Kurminas, Dagnė Apynytė, Rytis Masiliūnas, Aleksandra Ekkert, Jurgita Valaikienė

**Affiliations:** 1 Clinic of Neurology and Neurosurgery, Institute of Clinical Medicine, Faculty of Medicine, Vilnius University, LT-03101 Vilnius, Lithuania; dalius.jatuzis@santa.lt (D.J.); dagne.apynyte@gmail.com (D.A.); rytis.masiliunas@santa.lt (R.M.); doctor.ekkert@gmail.com (A.E.); jurgita.valaikiene@santa.lt (J.V.); 2 Department of Radiology, Nuclear Medicine and Medical Physics, Institute of Biomedical Sciences, Faculty of Medicine, Vilnius University, LT-03101 Vilnius, Lithuania; marius.kurminas@santa.lt

**Keywords:** acute ischemic stroke, thrombectomy, C-reactive protein, outcomes, mortality

## Abstract

*Background and Objectives*: This research was conducted to explore the associations between C-reactive protein (CRP) levels after acute ischemic stroke (AIS) due to emergent large vessel occlusion and treatment with thrombectomy and in-hospital functional outcomes. *Materials and Methods*: Serum CRP and other laboratory parameters were evaluated in individuals who experienced AIS and underwent thrombectomy upon admission to a comprehensive stroke center at Vilnius University Hospital Santaros Klinikos in 2022. In-hospital functional outcomes were assessed according to the modified Rankin Scale (mRS) and were considered poor in cases with mRS > 2 at discharge or in-hospital mortality. Multiple logistic regression was used to evaluate potential confounders. *Results*: The analysis encompassed 179 individuals (57.0% male and 43.0% female; mean (SD) age, 71.54 (11.32) years). Significantly higher median (interquartile range) CRP levels were detected in the poor-outcome group (4.47 (2.11–14.20) vs. 2.43 (1.37–6.25) mg/L; *p* = 0.042). Multiple analyses adjusted for important confounders during the hospital stay did not show any significant associations between CRP and the evaluated AIS outcomes. Poor outcomes at discharge were associated with detected infection (OR = 6.12; 95% CI: 2.22–16.86; *p* < 0.001), but in-hospital mortality was related to a higher NIH Stroke Scale score (OR = 1.12; 95% CI: 1.03–1.22; *p* = 0.010). A higher estimated glomerular filtration rate was associated with lower in-hospital mortality (OR = 0.97; 95% CI: 0.95–0.99; *p* = 0.013). *Conclusions*: No statistically significant associations were identified between CRP levels on admission and in-hospital functional outcomes in patients with AIS treated with thrombectomy.

## 1. Introduction

Acute ischemic stroke (AIS) due to emergent large vessel occlusion (ELVO), if not treated in a timely manner with vessel-patency-restoring treatment, is associated with a poor prognosis in up to two-thirds of all cases [1]. Current AIS treatment for ELVO, in which an endovascular approach through mechanical thrombectomy is applied, significantly improves outcome. However, restoring an occluded vessel lumen alone is insufficient, as one in two individuals remains disabled despite successful recanalization of the ELVO [2]. In addition to irreversible brain damage prior to recanalization, at least three groups of interacting conditions are responsible for poor outcomes in AIS. One is associated with unachieved patency of the opened arterial tree, including its distal branches (microvascular obstruction and reperfusion injury) [3], another comprises premorbid conditions [4], and the third involves evolving AIS-associated complications [5]. Worldwide efforts to understand the mechanisms of evolving AIS and develop ways to overcome these obstacles are ongoing [3].

One of the most important systems involved in AIS etiology, evolution, and post-stroke recovery is the immune system [6,7]. Its extensive range of active molecules and cells with proinflammatory and anti-inflammatory capabilities operate in the damaged brain [8] and through the brain–peripheral organ axis over the course of AIS [7,9,10]. While the harmful impact of inflammation becomes evident within the first few hours after AIS onset via ischemia–reperfusion effects [11], the negative effects of the immune system are associated with stroke-induced immunodepression, leading to an increased risk of infectious complications [12]. This significant role of inflammation is supported by promising data on the use of anti-inflammatory compounds in stroke, derived from experimental AIS models [13]. Furthermore, inflammation initiated during brain ischemia has positive effects on brain debris removal [8] and repair [14]. Given that this complex immune system behavior has not produced statistically significant results in clinical trials testing the use of inflammation-inhibiting substances in AIS [15,16], there is a need for a better understanding of the immune system components involved in AIS.

An active substance in the innate immune system, measured in everyday emergent stroke care clinics, is C-reactive protein (CRP). This protein is recognized as a “robust biomarker” for monitoring various inflammatory conditions [17]; it is potentially linked to thrombi that are more resistant to removal [18] and decreased activity of administered tissue plasminogen activator [19], and it aggravates the growth of infarcted brain tissue [20]. Research has established a positive association between early increased serum CRP levels after AIS and negative outcomes [21,22,23,24]. These associations could be explained by underlying conditions, including inflammatory vascular conditions that caused AIS, premorbid infections or neoplastic processes, and inflammation caused by ischemia-damaged brain tissue. However, conclusions regarding the relationship between CRP and AIS treated with reperfusion therapy are less clear. For example, in some AIS research involving thrombolysis, elevated CRP on admission was not associated with poor AIS outcomes, such as symptomatic intracranial hemorrhage (sIH), in-hospital and ninety-day mortality, the development of pneumonia [25], early neurological deterioration, or modified a Rankin Scale (mRS) > 2 after three months [26]. In contrast, other research found an association between elevated CRP and worse thrombolysis response [27] and three-month mortality [28]. Similar inconsistencies across studies are present in cases where AIS treatment with thrombectomy for ELVO has been applied. Some studies have found that increased baseline CRP is an independent predictor of poor outcomes defined as mRS > 2, long-term mortality [29], and neurological exacerbation at discharge [30], while others have not [31,32].

To further clarify these inconsistent findings, this study was conducted to evaluate the associations between routinely measured CRP at admission and discharge outcomes among patients with AIS undergoing mechanical clot removal. The discharge outcome data were selected because of the limited availability of longer-term outcome data.

## 2. Materials and Methods

### 2.1. Study Design, Population, and Outcomes

In this retrospective cohort study, we included adult patients (≥18 years old) with AIS due to ELVO of the anterior (internal carotid artery (ICA), middle cerebral artery (MCA) M1 and/or M2 and/or M3 segments) or posterior (posterior cerebral artery (PCA) P1 segment, basilar artery (BA), or vertebral artery (VA) V4 segment) circulation. AIS was confirmed by computed tomography (CT), CT angiography (CTA), CT perfusion, and digital subtraction angiography (DSA), according to the updated 21st-century definition of stroke [33]. Patients who fulfilled the European Stroke Organisation criteria for mechanical thrombectomy [34] were treated with thrombectomy; these patients were admitted to a single comprehensive stroke center at Vilnius University Hospital Santaros Klinikos over a one-year period in 2022. We excluded individuals who were unsuitable for thrombectomy because of (a) more than 24 h since the onset of symptoms; (b) brain hemorrhage and/or an Alberta Stroke Program Early CT (ASPECT) score < 6 and/or an extensive subtentorial lesion on the initial CT scan in posterior circulation AIS cases; (c) presentation with a non-disabling neurological deficit, as defined by the Potential of rtPA for Ischemic Strokes With Mild Symptoms (PRISMS) [35]; (d) premorbid disability according to the modified Rankin Scale (mRS) > 2; or (e) severe comorbidity with an estimated survival of <6 months.

Data collected on admission included demographic characteristics (age, sex, neurological impairment according to the NIH Stroke Scale (NIHSS)), conventional vascular risk factors (hypertension, diabetes mellitus (DM), atrial fibrillation (AF), previous stroke and/or myocardial infarction, peripheral artery disease, heart failure (HF) according to the New York Heart Association class score > 2, dyslipidemia with prescribed statins), and other premorbid factors (known malignancy). Routine blood samples were taken to measure leucocyte count, CRP, and creatinine on admission. Creatinine was used to calculate the estimated glomerular filtration rate (eGFR). We enrolled all AIS patients who received thrombectomy with or without intravenous thrombolysis during the study period (2022), regardless of their CRP values on admission, to maintain a larger number of participants.

Radiological data were collected, including the ASPECT score for anterior circulation and at least moderately expressed leukoencephalopathy according to Fazekas on the initial CT scan [36], the site of occlusion on CTA and DSA scans, successful recanalization according to a modified Treatment in Cerebral Infarction (mTICI) score ≥ 2c, and the rate of intracerebral hemorrhage after thrombectomy according to the European Cooperative Acute Stroke Study II criteria [37]. The subtypes of ELVO were categorized into large artery atherosclerosis (LAA), cardioembolic, and other according to the Trial of Org 10172 in Acute Stroke Treatment (TOAST) classification scheme [38]. In cases where two potential stroke etiologies were identified concurrently in the same individual (AF and LAA), LAA was selected as the underlying cause when the criteria of the European Society for Vascular Surgery 2023 Clinical Practice Guidelines on the Management of Atherosclerotic Carotid and Vertebral Disease were fulfilled [39]. The incidence of AIS complications during the hospital stay (bacterial infection, COVID-19 disease, venous thrombosis, pulmonary embolism, newly detected AF, delirium, and anxiety/depression) was evaluated. A bacterial respiratory tract infection was diagnosed according to appropriate symptoms and signs (new purulent sputum, cough, dyspnea, bronchial breath sounds, or altered mentality in older patients) and workup (fever, leukopenia or leukocytosis, and chest radiograph showing infiltrate and/or a positive respiratory secretion culture). A bacterial urinary tract infection was diagnosed according to positive appropriate symptoms and signs (new urinary retention or incontinence, dysuria or confusion in older patients) and workup (fever, leukopenia or leukocytosis, and positive urinalysis and urine culture for bacterial invasion). Other infections, such as COVID-19, sepsis, and endocarditis, were diagnosed according to local protocols.

The functional outcomes at discharge were dichotomized into (a) good (mRS ≤ 2) and poor (mRS > 2) and (b) survivors and non-survivors. Death was defined as all-cause mortality.

### 2.2. Statistical Analysis

IBM SPSS Version 29.0.0.0 (Chicago, IL, USA) was applied for statistical analysis [40]. CRP and other laboratory parameters were evaluated as continuous variables. Additionally, the participants were divided into those with normal CRP (≤5 mg/L) and increased CRP (>5 mg/L) according to local laboratory criteria and into those who experienced posterior or anterior circulation AIS.

Continuous data with a normal distribution (according to the Kolmogorov–Smirnov test) are reported as the mean ± standard deviation (SD), while other data are reported as medians with interquartile ranges (IQRs). Categorical variables were compared between groups using the Chi-square test with Fisher’s exact test (2-sided) and the Monte Carlo method for variables with >2 frequencies. Student’s *t*-test and the Mann–Whitney U test were used, as appropriate, for continuous variables. Variables that were significantly different between AIS outcome groups were included in a multiple logistic regression (LR) analysis to clarify the influence of confounders, and the results are reported as odds ratios (ORs), with 95% confidence intervals presented in parentheses for both univariate and multiple LR. Logistic regression analysis was performed twice, first with all variables available at admission (before thrombectomy) and then with all variables measured during the hospital stay. This approach assessed whether significant admission variables remained important predictors throughout the entire hospitalization period for AIS patients treated with thrombectomy. Finally, post hoc power analysis was performed using G*powers 3.1.9.6 for the 2 × 2 comparisons of normal CRP across both study outcomes, using Cohen’s w and the standard effect size for chi-square analysis [41]. Statistical tests were two-tailed, and *p* < 0.05 was considered significant.

## 3. Results

A total of 179 patients were included in this study. The mean age was 71.54 ± 11.32 years, and 57.0% of the individuals were male. The median NIHSS on admission was 9 (5–14). Normal CRP was found in 57.5% of participants, but the median CRP was 3.73 (1.53–12.45) mg/L. All other parameters and differences between the AIS outcome groups are presented in Table 1. Subjects with poor outcomes at discharge had more severe neurological impairment according to the NIHSS score (*p* = 0.002), a higher prevalence of previous HF (*p* = 0.047), higher median CRP values (*p* = 0.042), less frequent thrombolysis (*p* = 0.043), and a higher incidence of detected bacterial infection (*p* = 0.001). Patients who died during hospitalization had more severe neurological impairment according to the NIHSS score (*p* = 0.002), were more likely to have prior DM (*p* = 0.030), had a lower median eGFR (*p* = 0.004), were less likely to have received thrombolysis treatment (*p* = 0.043), and had more detected bacterial infections (*p* = 0.018). No differences were found in AIS outcomes between patients with normal and elevated CRP levels at admission.

No significant discrepancies were found between groups with normal versus elevated CRP levels regarding successful recanalization (*p* = 0.543) or hemorrhagic transformation (*p* = 0.489) (Supplementary Table S1).

A comparison of those who experienced anterior and those who experienced posterior AIS is provided in Supplementary Table S2. No significant differences were found between the groups.

The results of the applied LR are shown in Table 2. Univariate logistic regression analysis did not show an association between admission CRP and poor outcomes at discharge (OR = 1.01; 95% CI: 0.99–1.12; *p* = 0.124). After adjustment for significant variables identified in the univariate analysis between the good- and poor-outcome groups of AIS patients treated with thrombectomy (NIHSS, HF, and thrombolysis), elevated NIHSS scores remained significantly associated with poor discharge outcomes at admission (OR = 1.12; 95% CI: 1.04–1.20; *p* = 0.002), while thrombolysis was associated with lower odds of poor outcomes (OR = 0.39; 95% CI: 0.16–0.95; *p* = 0.038). Adjustment for all important variables during the hospital stay (NIHSS, HF, thrombolysis, and detected bacterial infection with 31.5 poor-outcome events per variable) identified detected bacterial infection (OR = 6.12; 95% CI: 2.22–16.86; *p* < 0.001) as the only independent predictor of poor prognosis.

After adjustment, significant variables identified on admission for predicting in-hospital mortality during the hospital stay (NIHSS, DM, and eGFR) remained associated with elevated NIHSS (OR = 1.13; 95% CI: 1.05–1.23; *p* = 0.002) and known DM (OR = 3.41; 95% CI: 1.16–10.01; *p* = 0.026), but a higher eGFR was associated with lower odds of death (OR = 0.97; 95% CI: 0.95–0.99; *p* = 0.009). Multiple LR analysis including all significant variables during the hospital stay (NIHSS, DM, eGFR, and detected bacterial infection, with just 5.5 death events per variable) revealed that only an elevated NIHSS score (OR = 1.12; 95% CI: 1.03–1.22; *p* = 0.010) remained independently associated with mortality, while a higher eGFR was associated with lower odds of death (OR = 0.97; 95% CI: 0.95–0.99; *p* = 0.013). Due to the limited number of events of in-hospital mortality a robustness analysis of the applied LR model was performed. Findings consistent with good model fit (Hosmer–Lemeshow test *p* = 0.674), no changes in conclusions after testing for outliers (without cases with Cook’s value > 1.0), and no multicollinearity (all tolerance values exceeded 0.30, and Variance Inflation Factor values ranged from 1.025 to 1.149) established that the applied LR was robust.

The post hoc power analysis of normal CRP levels comparing good versus poor outcomes corresponded to a small effect size (w = 0.11), but the comparison between survivors versus non-survivors showed an even smaller effect size (w = 0.09).

## 4. Discussion

In this single-center retrospective observational study, we found no statistically significant associations between admission CRP serum levels and poor outcomes at discharge or in-hospital mortality. The analysis included patients who experienced AIS secondary to ELVO and received thrombectomy. However, the results should be interpreted with caution, as the study was conducted at a single center and was underpowered due to the limited number of patients.

Our results are consistent with those of the MR CLEAN multicenter study, which examined AIS patients who underwent thrombectomy, had admission CRP ≤ 20 mg/L, and met the criteria for LAA- or AF-associated AIS. Using stricter inclusion criteria and a larger sample size compared to our study, the MR CLEAN multicenter study found no associations between CRP and AIS outcomes at 3 months, including mortality, successful recanalization, and the symptomatic intracranial bleeding rate [32].

Similar results regarding CRP were obtained in another single-center study, i.e., no significant associations were observed with the 3-month outcome in AIS patients treated with thrombectomy [31].

Contrary to our results, a study that included AIS secondary to internal carotid artery and MCA M1 occlusion found an association between increased CRP and worse discharge outcomes [30]; however, that study did not adjust for acquired infections.

Another study included anterior circulation AIS with applied thrombectomy and revealed an association between elevated CRP levels and poor clinical outcomes [29]. However, the researchers did not analyze the impact of infection on AIS outcomes. In our study, by comparison, one explanation for the lack of association between CRP and in-hospital outcomes could be the very early timing of the CRP test and CRP evaluation at a single time point.

This hypothesis is based on the results of a recent study that showed a significant association between serum CRP and poor prognosis after thrombectomy. That study used CRP concentrations measured 24 h post-thrombectomy [42]. Another study showed that, after AIS, the highest CRP concentration—especially in the context of an infection—was reached 5–7 days after the onset of AIS symptoms [43]. Another study analyzing serial CRP measurements showed that, during the first 24 h, CRP levels in individuals in whom an infection was identified upon hospitalization did not differ from those in whom no infectious disease was detected. However, a significant difference in CRP levels between the groups was observed starting from the third day of hospitalization [44]. Since the CRP response is not specific to inflammation of any origin, an elevation in CRP at the onset of an AIS may be associated with both risk factors related to the occurrence of AIS, such as atherosclerosis, and an inflammatory response to pre-existing or developing conditions, including those that are infectious [43]. Measuring additional inflammatory markers that markedly increase during the first 24 h, such as IL-6 [45], likely offers additional value in predicting AIS outcomes, including the risk of stroke-associated infection [46].

Independent of CRP levels, our study revealed, as expected, that acquired bacterial infection was the main variable associated with poor discharge outcomes. This finding aligns with evidence that up to one-third of all stroke-associated negative long-term consequences are related to infectious complications [12,47]. Experimental studies in mice have shown that, during bacterial infection in AIS, bacterial constituents, such as lipopolysaccharide or cytolysin, which induce a detrimental immune response, have the strongest negative effect [48,49]. Another possible lipopolysaccharide-associated negative effect on AIS outcomes could be related to autoimmune reactions against brain myelin basic protein [50]. It is also important to note that infectious complications typically develop more than 24 h after the onset of stroke symptoms, which could influence the CRP response and its potential association with consequent outcomes measured at later time points.

In our study, the key variables that remained predictive of lethal outcomes during hospitalization were a higher NIHSS and lower eGFR. As revealed in other studies, the severity of neurological impairment assessed by the NIHSS was recognized as a useful tool for stroke prognostication [51] because the NIHSS score significantly correlates with ischemic stroke volume [52] and the probability of large artery occlusion [53]. Moreover, the NIHSS score accounted for the largest proportion of variation in in-hospital mortality [54]. Furthermore, we found that a lower eGFR, a marker of renal impairment, was associated with in-hospital mortality after reperfusion therapy for AIS [55], which was also a risk factor for stroke [56] and linked to poor outcomes after stroke, including mortality [57]. The relationship between stroke and kidney injury is multifactorial and thought to be mediated through inflammation, multimorbidity, and aging [58].

It is important to understand that CRP is not toxic per se, as shown by studies conducted in volunteers [59]. To exert its function, CRP must be converted into active isoforms. In vivo, pentameric CRP (pCRP) undergoes conversion to modified pCRP under certain conditions, including ligand binding at regions of high membrane curvature and mildly acidic microenvironments, such as those present at sites of inflammation [60]. This fact could be associated with conflicting research results because active CRP isoforms are not specifically evaluated in routine practice. Furthermore, there is evidence that CRP can act as an anti-inflammatory substance [61]. Further research is needed to determine the significance of CRP in the prognosis of AIS patients treated with thrombectomy.

### Limitations

This study had the following limitations: (a) It included a limited number of participants from a single center, which carries a risk of false-negative results. (b) It evaluated only early CRP and did not address the dynamic inflammatory response. (c) The time of infection onset was not determined—that is, whether the infection began before or during hospitalization. (d) No data on other inflammation-associated biomarkers were collected. (e) A heterogeneous etiological classification of AIS was applied. (f) Information on stroke volume, collateral circulation, and perfusion mismatch in all patients was omitted. (g) There were no data on 90-day or longer-term outcomes of AIS.

## 5. Conclusions

Our study revealed no statistically significant association between admission CRP levels and in-hospital clinical outcomes in AIS patients treated with thrombectomy. These findings complement the results of other studies by showing that admission CRP is not associated with outcomes in ELVO stroke treated with endovascular therapy. Further multicenter research with serial CRP measurements together with more rigorous determination of the onset of infection, and a sample size sufficient to reach enough power for statistical generalization is warranted.

## Figures and Tables

**Table 1:** Characteristics of AIS patients treated with thrombectomy, categorized by discharge outcome status.

Characteristics	All AIS Patients	Outcomes at Discharge					
		Good	Poor	*p*	Survivors	Non-Survivors	*p*
n	179	53	126		157	22	
**Demographic**							
Age, years, mean ± SD	71.54 ± 11.32	69.77 ± 10.05	72.28 ± 11.77	0.085	70.99 ± 11.56	75.45 ± 8.62	0.083
Male, n (%)	102 (57.0)	36 (67.9)	66 (52.4)	0.069	87 (55.4)	15 (68.2)	0.358
Female, n (%)	77 (43.0)	17 (32.1)	60 (47.6)		70 (44.6)	7 (31.8)	
NIHSS, median (IQR)	9 (5–14)	7 (4–10)	10 (6–15)	**0.002**	8 (5–13)	14 (9–19)	**0.002**
**Known vascular risk factors**							
Hypertension, n (%)	139 (77.7)	37 (69.8)	102 (81.0)	0.118	122 (77.7)	17 (77.3)	1.000
DM, n (%)	31 (17.3)	7 (13.2)	24 (19.0)	0.394	23 (14.6)	8 (36.4)	**0.030**
AF, n (%)	55 (30.7)	18 (34.0)	37 (29.4)	0.596	51 (32.5)	4 (18.2)	0.221
Previous stroke, n (%)	24 (13.4)	9 (17.0)	15 (11.9)	0.350	22 (14.0)	2 (9.1)	0.743
Previous myocardial infarction, n (%)	18 (10.1)	4 (7.5)	14 (11.1)	0.592	16 (10.2)	2 (9.1)	1.000
Previous peripheral artery disease, n (%)	5 (2.8)	1 (1.9)	4 (3.2)	1.000	4 (2.5)	1 (4.5)	0.485
HF, n (%)	30 (16.8)	4 (7.5)	26 (20.6)	**0.047**	25 (15.9)	5 (22.7)	0.379
Dyslipidemia with prescribed statins	28 (15.6)	10 (18.2)	18 (15.0)	0.594	25 (16.0)	3 (15.8)	0.979
**Other premorbid factors affecting outcomes**							
Known malignancy, n (%)	22 (12.3)	8 (15.1)	14 (11.1)	0.462	20 (12.7)	2 (9.1)	1.000
**Laboratory findings on admission**							
Leucocyte (×10^9^/L), median (IQR)	7.99 (6.52–10.10)	7.99 (6.27–9.61)	8.06 (6.58–10.40)	0.212	7.99 (6.57–10.06)	8.21 (6.42–10.75)	0.600
CRP (mg/L), median (IQR)	3.73 (1.53–12.45)	2.43 (1.37–6.25)	4.47 (2.11–14.20)	**0.042**	3.51 (1.55–11.37)	6.27 (1.32–15.90)	0.619
CRP ≤ 5 mg/L, n (%)	103 (57.5)	35 (66.0)	68 (54.0)	0.185	93 (59.2)	10 (45.5)	0.254
eGFR (mL/min/1.73 m^2^), mean ± SD	71.90 ± 23.04	75.62 ± 20.67	70.33 ± 23.88	0.162	73.78 ± 23.14	58.64 ± 17.69	**0.004**
**Radiological findings on admission**							
ASPECTS, median (IQR) *	10 (8–10)	10 (9–10)	10 (8–10)	0.100	10 (8.75–10)	9.5 (8–10)	0.269
Leukoencephalopathy, n (%)	36 (20.1)	11 (20.8)	25 (19.8)	1.000	28 (17.8)	8 (36.4)	0.051
MCA M1 occlusion, n (%)	40 (22.3)	8 (15.1)	32 (25.4)	0.169	35 (22.3)	5 (22.7)	1.000
MCA M2 occlusion, n (%)	36 (20.1)	12 (22.6)	24 (19.0)	0.683	33 (21.0)	3 (13.6)	0.574
MCA M3 occlusion, n (%)	4 (2.2)	3 (5.7)	1 (0.8)	0.078	4 (2.5)	0 (0)	1.000
Carotid tandem occlusion, n (%)	33 (18.4)	10 (18.9)	23 (18.3)	1.000	29 (18.5)	4 (18.2)	1.000
Carotid occlusion, n (%)	38 (21.2)	12 (22.6)	26 (20.6)	0.766	30 (19.1)	6 (36.4)	0.064
BA occlusion, n (%)	6 (3.4)	1 (1.9)	5 (4.0)	0.671	5 (3.2)	1 (4.5)	0.550
VA V4 occlusion, n (%)	13 (7.3)	5 (9.6)	8 (6.3)	0.531	12 (7.6)	1 (4.5)	1.000
PCA P1 occlusion, n (%)	9 (5.0)	2 (3.8)	7 (5.6)	1.000	9 (5.0)	0 (0)	0.604
**Thrombolysis**							
Applied thrombolysis, n (%)	28 (15.6)	13 (24.5)	15 (11.9)	**0.043**	25 (16.0)	3 (13.6)	1.000
**Recanalization**							
mTICI = 2c or 3, n (%)	100 (55.9)	35 (66.0)	65 (51.6)	0.099	87 (55.4)	13 (59.1)	0.821
mTICI5 < 2c, n (%)	79 (44.1)	18 (34.0)	61 (48.4)		70 (44.6)	9 (40.9)	
**Post-thrombectomy radiological outcomes: hemorrhagic transformation**							
HI1, n (%)	36 (20.1)	9 (17)	27 (21.6)	0.823	29 (18.5)	7 (33.3)	0.101
HI2, n (%)	3 (1.7)	1 (1.9)	2 (1.6)		3 (1.9)	7 (33.3)	
PH1, n (%)	5 (2.8)	1 (1.9)	4 (3.2)		4 (2.5)	1 (4.8)	
PH2, n (%)	2 (1.1)	0 (0)	2 (1.6)		1 (0.6)	1 (4.8)	
**Ischemic stroke category**							
LAA, n (%)	66 (36.9)	20 (37.7)	46 (36.5)	0.955	56 (35.7)	10 (45.5)	0.457
Cardioembolism, n (%)	53 (29.6)	16 (30.2)	37 (29.4)		49 (31.2)	4 (18.2)	
Other, n (%)	60 (33.5)	17 (32.1)	43 (34.1)		52 (33.1)	8 (36.4)	
**Non-neurological complications**							
Bacterial infection, n (%)	67 (37.4)	6 (11.3)	61 (48.8)	**<0.001**	54 (34.4)	13 (61.9)	**0.018**
COVID-19 disease, n (%)	12 (6.7)	3 (5.5)	9 (7.3)	0.647	10 (6.3)	2 (10.5)	0.486
Venous thrombosis, n (%)	8 (4.5)	2 (3.8)	6 (4.8)	1.000	8 (5.1)	0 (0)	0.599
Pulmonary embolism, n (%)	2 (1.1)	0 (0)	2 (1.6)	1.000	2 (1.3)	0 (0)	1.000
Anemia requiring blood transfusion, n (%)	6 (3.4)	2 (3.8)	4 (3.2)	1.000	6 (3.8)	0 (0)	1.000
Newly detected AF, n (%)	27 (15.1)	6 (11.3)	21 (16.7)	0.493	25 (15.9)	2 (9.1)	0.536
**Neuropsychiatric complications**							
Delirium, n (%)	11 (6.1)	1 (1.9)	10 (7.9)	0.178	11 (7.0)	0 (0)	0.364
Anxiety/depression, n (%)	5 (2.8)	0 (0)	5 (4)	0.324	5 (3.2)	0 (0)	1.000

AF, atrial fibrillation; AIS, acute ischemic stroke; ASPECTS, Alberta stroke program early CT score; BA, basilar artery; CRP, C-reactive protein; DM, diabetes mellitus; eGFR, estimated glomerular filtration rate; HF, heart failure; HI1, hemorrhage infarction type 1; HI2, hemorrhage infarction type 2; IQR, interquartile range; LAA, large artery atherosclerosis; MCA, middle cerebral artery; mTICI, modified Treatment in Cerebral Infarction score; NIHSS, National Institutes of Health Stroke Scale; PCA, posterior cerebral artery; PH1, parenchymal hematoma type 1; PH2, parenchymal hematoma type 2; SD, standard deviation; VA, vertebral artery. Numbers in bold indicate significant differences, *p* < 0.05. * ASPECTS score attributed solely to an anterior circulation AIS (n = 151).

**Table 2:** Logistic regression analysis of associations between clinical variables and poor outcomes at discharge.

Parameters	Univariate			Multiple		
	OR	95%CI	*p*	OR	95%CI	*p*
**Variables on admission for prediction of bad outcomes at discharge**						
NIHSS	1.11	1.04–1.18	**0.003**	1.12	1.04–1.20	**0.002**
HF	3.18	1.05–9.63	**0.047**	2.31	0.73–7.38	0.156
CRP	1.01	0.99–1.12	0.124	1.01	0.96–1.01	0.201
Thrombolysis	0.41	0.18–0.93	**0.041**	0.39	0.16–0.95	**0.038**
**Variables during the hospital stay for prediction of bad outcomes at discharge**						
NIHSS	1.11	1.04–1.18	**0.003**	1.08	1.00–1.16	0.52
HF	3.18	1.05–9.63	**0.047**	2.85	0.85–9.59	0.09
CRP	1.01	0.99–1.12	0.124	1.00	0.99–1.01	0.972
Thrombolysis	0.41	0.18–0.93	**0.041**	0.402	0.16–1.02	0.055
Detected bacterial infection	7.47	2.98–18.72	**<0.001**	6.12	2.22–16.86	**<0.001**
**Variables on admission for in-hospital mortality prediction**						
NIHSS	1.13	1.05–1.22	**0.001**	1.13	1.05–1.23	**0.002**
DM	3.33	1.26–8.82	**0.030**	3.41	1.16–10.01	**0.026**
eGFR	0.97	0.95–0.99	**0.005**	0.97	0.95–0.99	**0.009**
**Variables during the hospital stay for in-hospital mortality prediction**						
NIHSS	1.13	1.05–1.22	**0.001**	1.12	1.03–1.22	**0.010**
DM	3.33	1.26–8.82	**0.030**	2.69	0.87–8.37	0.087
eGFR	0.97	0.95–0.99	**0.005**	0.97	0.95–0.99	**0.013**
Detected bacterial infection	3.10	1.21–7.94	**0.018**	1.82	0.61–5.44	0.286

CRP, C-reactive protein; DM, diabetes mellitus; eGFR, estimated glomerular filtration rate; HF, heart failure; NIHSS, National Institutes of Health Stroke Scale. Numbers in bold indicate significant differences, *p* < 0.05.

## Data Availability

The raw data supporting the conclusions of this article will be made available by the authors, without undue reservation, upon reasonable request.

## Supplementary Materials

The following supporting information can be downloaded at https://www.mdpi.com/article/10.3390/medicina62081462/s1.

## Abbreviations

The following abbreviations are used in this manuscript:

AF	Atrial fibrillation
AIS	Acute ischemic stroke
ASPECTS	Alberta stroke program early CT score
BA	Basilar artery
CRP	C-reactive protein
DM	Diabetes mellitus
eGFR	Estimated glomerular filtration rate
HF	Heart failure
HI1	Hemorrhage infarction type 1
HI2	Hemorrhage infarction type 2
IQR	Interquartile range
LAA	Large artery atherosclerosis
MCA	Middle cerebral artery
mRS	Modified Rankin Scale
mTICI	Modified Treatment in Cerebral Infarction Score
NIHSS	National Institutes of Health Stroke Scale
PCA	Posterior cerebral artery
PH1	Parenchymal hematoma type 1
PH2	Parenchymal hematoma type 2
SD	Standard deviation
VA	Vertebral artery
