# Supplementary material for: Routine C-Reactive Protein on Admission and In-Hospital Outcomes in Acute Ischemic Stroke Patients Treated with Thrombectomy: A Retrospective Single-Center Study
Source: Medicina (Kaunas). 2026 Jul 28;62(8):1462. doi: 10.3390/medicina62081462 (PMC13515061; doi:10.3390/medicina62081462)
Supplement: Supplementary file 1 [file medicina-62-01462-s001.zip › medicina-4395727-supplementary.pdf]

Supplementary Materials

Supplementary Table S1. Successful recanalization and any hemorrhagic transformation by CRP levels.

**Characteristics**	**CRP ≤ 5 mg/L (n = 103)**	**CRP > 5 mg/L (n = 76)**	**OR**	**95% CI**	***p***
mTICI = 2c or 3, n (%)	60 (41.7)	40 (52.6)	1.26	0.69-2.28	0.543
Haemorrhagic transformation, n (%)	24 (23.3)	22 (28.9)	1.34	0.68-2.63	0.489

CRP, C-reactive protein; mTICI, modified Treatment in Cerebral Infarction Score.

Supplementary Table S2. Characteristics of AIS patients treated with thrombectomy, categorized by posterior circulation infarction and anterior circulation infarction.

**Characteristics**	**Posterior circulation (n = 28)**	**Anterior circulation (n = 151)**	**p**
**Demographic**			
Age, years, mean ± SD	70.14 ± 9.32	71.79 ± 11.66	0.480
Male, n (%)	20 (71.4)	82 (54.3)	0.101
Female, n (%)	8 (28.6)	69 (45.7)	
NIHSS, median (IQR)	7 (4-12)	9 (6-14)	0.261
**Known vascular risk factors**			
Hypertension, n (%)	21 (75.0)	118 (78.1)	0.805
DM, n (%)	3 (10.7)	28 (18.5)	0.420
AF, n (%)	10 (35.7)	45 (29.8)	0.514
Previous stroke, n (%)	4 (14.3)	20 (13.2)	1.000
Previous myocardial infarction, n (%)	1 (3.6)	17 (11.3)	0.315
Previous peripheral artery disease, n (%)	0 (0)	5 (3.5)	1.000
HF, n (%)	2 (7.1)	28 (18.5)	0.175
Dyslipidemia with prescribed statins	4 (14.3)	23 (15.2)	0.900
**Other premorbid factors affecting outcomes**			
Known malignancy, n (%)	2 (7.1)	20 (13.2)	0.535
**Laboratory findings on admission**			
Leucocyte (x 10^9/L), median (IQR)	8.93 (6.71-10.24)	7.90 (6.42-10.02)	0.258
CRP (mg/L), median (IQR)	2.66 (1.46-4.95)	4.18 (1.6-14.02)	0.117
CRP ≤ 5 mg/L, n (%)	21 (75.0)	82 (54.3)	0.060
eGFR (mL/min/1.73 m^2), mean ± SD	75.04 ± 23.29	71.32 ± 23.03	0.435
**Radiological findings on admission**			
Leukoencephalopathy, n (%)	2 (7.1)	34 (22.5)	0.074
**Thrombolysis**			
Applied thrombolysis, n (%)	5 (17.9)	23 (15.3)	0.778
**Recanalization**			
mTICI = 2c or 3, n (%)	14 (50.0)	86 (57.0)	0.538
mTICI5 < 2c, n (%)	14 (50.0)	65 (43.0)	
**Post-thrombectomy radiological outcomes: hemorrhagic transformation**			
HI1, n (%)	5 (17.9)	31 (20.7)	0.486

HI2, n (%)	0 (0)	3 (2.0)	
PH1, n (%)	0 (0)	5 (3.3)	
PH2, n (%)	1 (3.6)	1 (0.7)	
**Ischemic stroke category**			
LAA, n (%)	9 (32.1)	57 (37.7)	0.272
Cardioembolism, n (%)	6 (21.4)	47 (31.1)	
Other, n (%)	13 (46.4)	47 (31.1)	
**Non-neurological complications**			
Bacterial infection, n (%)	10 (35.7)	57 (38.0)	1.000
COVID-19 disease, n (%)	0 (0)	12 (8.0)	0.218
Venous thrombosis, n (%)	1 (3.6)	7 (4.7)	1.000
Pulmonary embolism, n (%)	0 (0)	2 (1.3)	1.000
Anemia requiring blood transfusion, n (%)	1 (0)	6 (4.0)	0.592
Newly detected AF, n (%)	4 (14.3)	23 (15.2)	1.000
**Neuropsychiatric complications**			
Delirium, n (%)	3 (10.7)	8 (5.3)	0.382
Anxiety / depression, n (%)	1 (3.6)	4 (2.6)	0.577
**Outcomes at discharge**			
Poor, n (%)	20 (71.4)	106 (70.2)	1.000
In-hospital mortality, n (%)	2 (7.1)	20 (13.2)	0.535

AF, atrial fibrillation; AIS, acute ischemic stroke; CRP, C-reactive protein; DM, diabetes mellitus; eGFR, estimated glomerular filtration rate; HF, heart failure; HI1, hemorrhage infarction type 1; HI2, hemorrhage infarction type 2; IQR, interquartile range; LAA, large artery atherosclerosis; mTICI, modified Treatment in Cerebral Infarction Score; NIHSS, National Institutes of Health Stroke Scale; PH1, parenchymal hematoma type 1; PH2, parenchymal hematoma type 2; SD, standard deviation.
